# Osteomyelitis and Its Main Determinants in Patients With Diabetic Foot Ulcer: A Cross‐Sectional Study

**DOI:** 10.1002/hsr2.71463

**Published:** 2025-11-09

**Authors:** Soheil Bolandi, Mohammad Hadi Niyakan, Kazem Jamali, Yasaman Ghodsi Boushehri, Ali Tajaddini, Reza Shahriarirad

**Affiliations:** ^1^ Department of Surgery Shiraz University of Medical Sciences Shiraz Iran; ^2^ Trauma Research Center, Shahid Rajaee Hospital Shiraz University of Medical Science Shiraz Iran; ^3^ Thoracic and Vascular Surgery Research Center Shiraz University of Medical Sciences Shiraz Iran

**Keywords:** deep tissue infection, diabetic foot ulcer, epidemiology, osteomyelitis

## Abstract

**Background and Aims:**

Diabetic foot ulcers (DFUs) represent a significant and prevalent complication of diabetes, imposing substantial psychological, financial, and healthcare burdens globally. Osteomyelitis, the most common complication of DFUs, is a primary cause of lower limb amputations in diabetic patients. This cross‐sectional study aimed to evaluate the epidemiological and clinical aspects of patients with diabetic foot osteomyelitis (DFOM) resulting from DFU infection.

**Methods:**

Hospital records with a diagnosis of diabetic foot wound infection were reviewed during a period of 8 months (April–December 2021). Osteomyelitis was diagnosed using magnetic resonance imaging criteria. Patients were categorized into two groups of with and without osteomyelitis, and compared accordingly for predisposing risk factors and also clinical outcome.

**Results:**

Among 252 cases of DFU infection, 23.8% were confirmed to have DFOM. Multivariate logistic regression analysis identified lower age (*p* = 0.02; OR = 0.86; 95%CI: 0.75–0.98), lower BMI (*p* = 0.01; OR = 0.57; 95%CI: 0.382–0.86), higher income level (*p* < 0.001; OR = 8.62; 95%CI: 2.42–30.74), higher HbA1c level (*p* < 0.001; OR = 19.072; 95%CI: 7.61–47.78), and the location of the wound on the heel and leg as predictive factors for the occurrence of DFOM. Regarding the clinical outcomes, osteomyelitis was significantly associated with longer length of hospitalization (19.02 ± 2.58 vs. 14.53 ± 2.74 days), delayed wound healing (above 6 months: 40.0 vs. 8.8%), lower limb salvage (68.3 vs. 99.0%), higher rate of wound dehiscence (33.3 vs. 10.4%), and higher rate of wound recurrence (30.0 vs. 0.5%). The mortality rate was also higher in the osteomyelitis group (13.3 vs. 0%).

**Conclusion:**

Approximately 23.8% of patients with DFU may develop osteomyelitis, which can be predicted by lower age, lower BMI, higher income level, higher HbA1c level, and the location of the wound. Early identification and appropriate management of these risk factors may help prevent osteomyelitis development in DFU patients.

## 1 Introduction

Diabetic foot ulcers (DFUs) are a common complication of diabetes caused by diabetic neuropathy, which can have significant psychological and financial impacts on patients, their families, and healthcare systems [1]. Past studies have shown that limb amputation is performed every 30 s due to DFUs [2]. The chronic course of diabetes can lead to poor blood supply caused by the deposition of glucose and glucose‐related metabolites in the small vessels of the terminal blood supply, causing slow and difficult wound healing [3]. DFUs account for more than 20% of hospitalizations due to diabetes and can lead to organ damage, deadly infections, and increased risk of lower limb amputation [4, 5]. Preventive measures and antibiotic treatment are critical in reducing the burden of DFUs, as about 60% of DFUs are complicated by infections, and infections are the leading cause of lower limb amputations in diabetic patients with diabetic feet [6, 7, 8, 9].

Diabetic foot wound infections are associated with serious morbidity, reduced quality of life, frequent hospitalizations, and even death, with nephropathy or ischemia being common underlying causes [10, 11]. Peripheral vascular disease and infection increase the risk of non‐healing and amputation [12].

Osteomyelitis, which is the most common target of diabetic foot wound infection, can occur in 10%–15% of moderate cases and 50% of severe cases of infection [13]. Wounds complicated by osteomyelitis often require surgical treatment and long‐term antibiotic therapy [14, 15, 16]. Osteomyelitis is associated with a high risk of amputation and typically occurs due to incurable wounds [17, 18, 19]. Diabetic foot osteomyelitis (DFOM) usually occurs as a result of a soft tissue infection that spreads to the bones, mainly affecting the bone cortex and sometimes the bone marrow. The possibility of bone involvement should always be considered in patients with DFUs with findings in favor of infection, especially in chronic diabetic ulcers and cases with ulcer recurrence.

Given the high prevalence of diabetes in Iran and the high number of DFUs, this study aims to investigate the prevalence and characteristics of patients with DFUs who have osteomyelitis and compare them to those without osteomyelitis.

## 2 Materials and Methods

A cross‐sectional study was conducted on patients with DFUs who were admitted to Namazi Hospital, Shiraz, Iran between April and December 2021 (8 months). Patients with ulcers of vascular origin were excluded, while traumatic diabetic ulcers were included in the study. Patients' hospital records were retrieved based on the special code of the disease, and data including demographic information, socioeconomic characteristics, place of residence, duration and hospitalization, type of lesion, previous history of the wound, as well as the need for amputation or debridement in osteomyelitis, and the level of amputation were collected and included in the forms prepared for this purpose. Patients were then divided into two groups based on the presence of clinical and imaging evidence of osteomyelitis.

Magnetic resonance imaging (MRI) criteria were used to diagnose osteomyelitis, following clinical suspicion, which included a focal decrease in bone marrow signal intensity (SI) in T1‐W images compared to normal bone marrow areas, a focal increase in SI of bone or bone marrow in fat‐covered T2‐W images relative to areas of normal bone or bone marrow, or focal enhancement of bone or bone marrow on contrast‐enhanced images (when adjacent secondary findings of cellulitis, soft tissue abscess, or sinus tract were present for these three criteria) or presence of bone destruction in T1‐W or T2‐W images. Findings that ruled out the diagnosis of osteomyelitis included SI of bone and normal bone marrow on both fat‐suppressed T1‐W and T2‐W images, whereas the presence of osteomyelitis was considered indeterminate in the presence of normal bone marrow SI on T1‐W images with increased SI on T2‐W images with or without secondary findings associated with soft tissue infection. Although bone biopsy is considered the gold standard for confirming osteomyelitis, it was not routinely performed, as per institutional clinical practice. MRI was used as the preferred diagnostic modality in cases with clinical and radiographic suspicion, following standard care protocols.

All ulcers undergo assessment by a surgeon. Wounds were graded by measuring their depth using a sterile blunt probe, and the deepest tissue involved was documented (dermis as grade 1, subcutaneous as grade 2, fascia as grade 3, muscle as grade 4, and bone as grade 5). A diagnosis of soft tissue infection was made if a purulent discharge was present along with two other local signs (such as warmth, erythema, lymphangitis, lymphadenopathy, edema, or pain). Locations of ulcers were categorized as follows: toes, big toe, metatarsals, midfoot/dorsum, heel/leg.

The management of DFU patients in our center is based on a multi‐disciplinary approach. For patients showing evidence of arterial insufficiency, we recommend referral to a vascular specialist, which performed additional evaluations and color doppler sonography. All patients received antibiotic therapy targeting the most common infecting organisms, which primarily included aerobic gram‐positive cocci [20]. Other frequent pathogens consisted of aerobic gram‐negative bacilli and anaerobes. A second empirical antimicrobial agent was selected based on infection severity and the likelihood of microbial resistance. Antibiotics were determined based on a standardized protocol developed by the Department of Infectious Diseases at Shiraz University of Medical Sciences. This protocol is updated annually and every 5 years based on local data on the most common infectious organisms and their antibiograms. Decisions were made collaboratively by an infectious disease specialist and the treating physician, ensuring region‐specific, evidence‐based, and targeted antibiotic therapy [20]. Additionally, comprehensive care included glycemic control with appropriate and routine nutrition and medications, fluid and electrolyte balance maintenance, and local wound treatment.

Local ulcer care involves attention to wound management, including callus and necrotic tissue debridement, wound cleansing, and pressure relief on the ulcer site. Ulcers graded 2 and above undergo sharp debridement and receive appropriate wound coverage. Surgical (sharp) debridement was performed following a standardized protocol, with wound assessments conducted every 3–5 days to determine the need for further intervention. Dressings were chosen based on ulcer appearance and characteristics, like amount of exudate, condition of the skin around the wound and complemented by offloading techniques such as total contact casts or specialized footwear to minimize pressure on the ulcer site. In cases of extensive open wounds following infection and necrosis debridement or partial foot amputation, we employ negative pressure wound therapy for wound management.

Statistical analysis was conducted using SPSS version 26.0 (IBM, Armonk, New York). Results were presented as mean ± standard deviation (SD) for quantitative variables and frequency (percentage) for categorical variables. Continuous variables were compared using t‐test or Mann‐Whitney test whenever the data did not appear to have normal distribution or when the assumption of equal variances was violated across the study groups. The main determinants for the presence of osteomyelitis were identified by multivariable logistic regression modeling with the presence of baseline parameters as the covariates. *p* values less than 0.05 were considered statistically significant.

## 3 Results

In this study, we investigated the prevalence and clinical outcomes of osteomyelitis in 252 cases of diabetic foot wound infection during our 8‐month study period. We found that 23.8% of cases had confirmed osteomyelitis. The features of our patients are demonstrated in Table 1. Furthermore, the MRI and wound images of the patients in our study are provided in Supporting Figures [Link], [Link], [Link], [Link].

**Table 1:** Baseline and clinical characteristics in patients with and without osteomyelitis.

Characteristics			Total; *N* = 252	Osteomyelitis		*p* value
				Positive; *n* = 60	Negative; *n* = 192	
**Age** (years); mean ± SD			60.11 ± 3.74	59.32 ± 3.90	60.35 ± 3.66	0.06
**Gender**, *n* (%)		Male	144 (57.1)	45 (75.0)	99 (51.6)	**0.001**
		Female	108 (42.9)	15 (25.0)	93 (48.4)	
**Body mass index** (kg/m^2^); mean ± SD			27.08 ± 1.41	26.87 ± 1.30	27.14 ± 1.43	0.19
**Education level**; *n* (%)		Undergraduate	54 (21.4)	13 (21.7)	41 (21.4)	0.89
		Diploma	188 (74.6)	44 (73.3)	144 (75.0)	
		Academic degree	10 (4.0)	3 (0.5)	7 (3.6)	
**Income status**; *n* (%)		Low	37 (14.7)	5 (8.3)	32 (16.7)	0.07
		Moderate	189 (75.0)	45 (75.0)	144 (75.0)	
		High	26 (10.3)	10 (16.7)	16 (8.3)	
**Location of wound**; *n* (%)		Small toes	123 (48.8)	13 (21.7)	110 (57.3)	**< 0.001**
		Big toe	37 (14.7)	10 (16.7)	27 (14.1)	
		Metatarsals	44 (17.5)	11 (18.3)	33 (17.2)	
		Midfoot/dorsum	34 (13.5)	14 (23.3)	20 (10.4)	
		Heel/leg	14 (5.6)	12 (20.0)	2 (1.0)	
**Hemoglobin A1c level** (%); mean ± SD			6.8 ± 1.0	7.2 ± 1.1	5.8 ± 0.7	**< 0.001**
**Mean length of hospitalization** (days); mean ± SD			15.6 ± 3.3	19.0 ± 2.6	14.5 ± 2.7	**< 0.001**
**Time of wound healing**; *n* (%)		< 3 months	95 (37.7)	4 (6.7)	91 (47.4)	**< 0.001**
		3–6 months	116 (46.0)	32 (53.3)	84 (43.8)	
		7–12 months	32 (12.7)	16 (26.7)	16 (8.3)	
		> 12 months	9 (3.6)	8 (13.3)	1 (0.5)	
**Limb salvage**			231 (91.7)	41 (68.3)	190 (99.0)	**< 0.001**
**Needing amputation**; *n* (%)			54 (21.4)	15 (25.0)	39 (20.3)	0.44
**Level of amputation**; *n* (%)		Over the knee	11 (4.4)	6 (10.0)	5 (2.6)	**< 0.001**
		Under the knee	44 (17.5)	10 (16.7)	34 (17.7)	
		Lower sites/other zones	38 (15.1)	18 (30.0)	20 (10.4)	
**Wound dehiscence**; *n* (%)			77 (30.6)	53 (88.3)	24 (12.5)	**< 0.001**
**Wound recurrence**; *n* (%)			19 (17.5)	18 (30.0)	1 (0.5)	**< 0.001**
**Death**; *n* (%)			8 (3.2)	8 (13.3)	0 (0)	**< 0.001**

*Note:* Bold values indicate significant association.

We evaluated the baseline features of the patients in our study based on the multivariate logistic regression model, to detect risk factors for osteomyelitis. Our model identified lower age (*p* = 0.02; OR = 0.86; 95%CI: 0.75 – 0.98), lower BMI (*p* = 0.008; OR = 0.57; 95%CI: 0.38 – 0.86), higher income level (*p* < 0.001; OR = 8.62; 95%CI: 2.42 – 30.74), higher HbA1c level (*p* < 0.001; OR = 19.07; 95%CI: 7.61 – 47.78), and the location of the wound as predictive factors for the occurrence of osteomyelitis. Gender and education did not achieve statistical significance in our model (Male vs. Female: *p* = 0.36; OR = 1.703; 95%CI: 0.19 – 1.84 and higher education: *p* = 0.12; OR = 0.40; 95%CI: 0.54 – 5.34).

Regarding the location of wound, wounds on the heel and leg had a significantly higher risk of osteomyelitis compared to other locations (*p* = < 0.001, < 0.001, < 0.001, 0.003 and OR = 0.002, 0.01, 0.001, 0.035 for small toes, big toe, metatarsal, midfoot/dorsum, respectively), while small toe wounds had a significantly lower risk of osteomyelitis compared to other locations except metatarsals (*p* = 0.03, 0.44, < 0.001, < 0.001 and OR = 5.32, 0.50, 14.62, 441.90 for big toe, metatarsal, midfoot/dorsum, and heel/leg respectively).

Regarding clinical outcomes (Table 1), osteomyelitis was significantly associated with longer length of hospitalization (19.02 ± 2.58 vs. 14.53 ± 2.74 days), delayed wound healing (above 6 months: 40.0 vs. 8.8%), lower limb salvage (68.3 vs. 99.0%), higher rate of wound dehiscence (33.3 vs. 10.4%), and higher rate of wound recurrence (30.0 vs. 0.5%). The mortality rate was also higher in the osteomyelitis group (13.3 vs. 0%). Higher need for amputation was also observed in the osteomyelitis group, although not significant (25.0 vs. 20.3%; *p* = 0.44).

## 4 Discussion

The occurrence of foot wound infection in patients with diabetes, especially uncontrolled diabetes mellitus, is a complication with significant morbidity and mortality. With the development of effective and broad‐spectrum antibiotics, this complication has been significantly improved and the complications caused by it have been minimized [21, 22]. However, a significant part of these patients may also be faced with infectious involvement of deeper tissues such as bones and joints, and therefore, the occurrence of osteomyelitis in such patients is not far from expected [23]. Therefore, numerous studies been developed and presented to prevent and reduce the occurrence of this complication and improve the clinical quality of life of patients. To achieve better‐desired outcomes in these patients, it seems necessary to evaluate the various epidemiological and clinical dimensions of the patients and find the risk factors related to its occurrence and exacerbation in every society. Our study suggests that approximately 23.8% of patients with DFUs may develop osteomyelitis, which can be predicted by lower age, lower BMI, higher income level, higher HbA1c level, and the location of the wound. Early identification and appropriate management of these risk factors may help prevent the osteomyelitis development in DFU patients.

Concerning the prevalence of osteomyelitis, a diverse spectrum of patients with diabetic foot wound infections experiences this complication, with variations primarily attributed to the diagnostic methods employed. This discrepancy is further influenced by demographic characteristics, patient selection criteria, and variations in the follow‐up duration. In our study, 60 out of 252 patients (23.8%) confirmed DFOM through clinical and MRI assessments. A 2015 UK study reported a higher prevalence, with 116 out of 275 patients (42%) diagnosed with DFOM using a combination of clinical, microbiological, and radiological criteria, primarily X‐rays [24]. In a study conducted by Andrianaki et al from 2011 to 2014 in Greece, osteomyelitis associated with diabetic foot or DFOM was confirmed in 18.1% (15/83) of patients [25]. Crisologo et al found in their study that 24 out of 35 patients (68.6%) developed osteomyelitis based on bone culture and histology, with 16.7% experiencing recurrent osteomyelitis [26]. The variability in diagnostic methods, particularly in defining the gold standard for diabetic foot infections, may contribute to the observed differences in osteomyelitis prevalence across studies [27]. Additionally, population‐specific variations in the prevalence of risk factors, such as diabetes, can also be a contributing factor [28]. The wide range of osteomyelitis prevalence in DFU patients underscores the complexity of its diagnosis and the need for standardized criteria. Addressing these variations is crucial for a comprehensive understanding of osteomyelitis in diabetic populations, aiding in the development of effective preventive and therapeutic strategies.

The predominant site for OM occurrence in our study was the midfoot and dorsum, followed by small toes. Previous studies have reported the forefoot to be the most predominant location for OM [24, 29, 30]. These results are unsurprising given that the majority of ulcers develop on the plantar surface, typically beneath the metatarsal heads or on the toes. Plantar ulcers, in particular, tend to emerge at locations experiencing elevated plantar pressure [31]. This is also concurrent with our findings demonstrating the most prevalent location of DFUs to be on the small toes (*n* = 123; 48.8%), followed by the metatarsals (*n* = 44; 17.5%). However, based on our regression model, wounds on the small toe had a significantly lower risk of osteomyelitis compared to other locations except metatarsals, while heel and leg wounds had a significantly higher risk of osteomyelitis compared to other locations. Notably, a study in the UK reported that though hindfoot wounds were relatively rare (3.6%), they exhibited a poor prognosis. The majority of these patients underwent surgical interventions, with outcomes often culminating in either above or below knee amputations, resulting in compromised mobility status—many becoming bedbound or reliant on wheelchairs [24]. For foot lesions involving the midfoot or heel, MRI is consistently necessary for confirmation [32]. Faglia et al., reported that having osteomyelitis localized in the heel significantly increases the risk of whole‐foot amputation. Primary amputations were exclusively conducted in cases where osteomyelitis was confined to the heel, as the spread of the infection precluded a partial calcanectomy [33]. Elevated amputation rates following partial calcanectomy underscore commitment to preserving the extremity, even when employing approaches associated with a high risk of failure [33, 34, 35]. Similar to our study, Faglia et al reported that the occurrence of osteomyelitis in the midfoot presents a lower risk of amputation above the ankle compared to the heel; however, the risk is considerably higher than that seen in patients with osteomyelitis of the forefoot [33].

The evaluation of DFOM outcomes can be evaluated through parameters such as the duration of hospitalization, ulcer healing time, and the occurrence of amputations [36]. The mean length of hospital stay in our study was significantly higher in DFOM compared to patients without DFOM (19.02 ± 2.58 vs. 14.53 ± 2.74 days). Previous studies which reported a hospitalization duration of around 3 weeks [24, 30]. Over 67% of patients were hospitalized for up to a month, and nearly 30% stayed between 1 and 3 months, constituting approximately 97% of the patient cohort [24]. However, in some studies such as Wukich et al, the average length of hospital stay was 7 days [37]. Although this study did not delve into the correlation between hospital stay duration and comorbidities, DFU severity, or the necessity for prolonged intravenous antibiotic therapy, these aspects warrant further investigation. The extended hospitalization of these patients poses a socioeconomic burden on both the healthcare system and the patients and their families. Therefore, exploring strategies to reduce this hospitalization period would undoubtedly alleviate this burden [38].

Regarding clinical outcomes, osteomyelitis was significantly associated delayed wound healing (above 6 months: 40.0 vs. 8.8%), lower limb salvage (68.3 vs. 99.0%), higher rate of wound dehiscence (33.3 vs. 10.4%), higher rate of wound recurrence (30.0 vs. 0.5%), and also higher mortality rate (13.3 vs. 0%). Higher need for amputation was also observed in the osteomyelitis group, although not significant (25.0 vs. 20.3%). In the study of Wukich et al, major amputation was done in 16.7% of cases with DFOM [37]. In the study of Crisologo et al, the cases of non‐healing of the wound in 8.3%, re‐ulceration in 20.8%, readmission in 16.7% and amputation in 12.5% were reported (20). In the study of Widatalla et al, initial wound healing less than 6 months was reported in 73% of patients following treatment, but amputation occurred in 15% of patients with osteomyelitis and 12% of patients with osteomyelitis had wound recurrence during the follow‐up period [39]. Arias et al reported that merely 2% of patients witnessed their wounds healing within a month, while 36% required 3–12 months for healing [24]. In comparison, the Eurodiale study group reported 23% of wounds remaining unhealed after 1 year of follow‐up [40]. During the first 6 months in our study, wound healing was achieved 60% of osteomyelitis cases compared to 91.2% without osteomyelitis. It seems that the guidelines and protocols used to prevent complications caused by diabetic foot wound infection, especially osteomyelitis, were very different in various societies, and therefore, the distribution of clinical outcomes was completely different.

According to the 2023 IWGDF guidelines, the diagnosis of diabetic foot osteomyelitis should be based on a combination of clinical, imaging, and microbiological findings. MRI is recommended as the most accurate imaging modality, particularly in cases where plain radiography is inconclusive. While bone biopsy remains the diagnostic gold standard, it is often reserved for cases where the diagnosis is uncertain or where microbiological confirmation is required to guide targeted therapy. The guidelines also emphasize the importance of managing DFOM within a multidisciplinary team setting, involving infectious disease specialists, radiologists, surgeons, and wound care experts. Our diagnostic approach and study design were aligned with these recommendations, particularly in the use of MRI and collaborative clinical decision‐making, though bone biopsy was not routinely performed due to institutional practice standards [41]. However, differences in patient populations, diagnostic criteria, and treatment protocols may cause discrepancies which warrant further investigation. Adhering to regional or international guidelines can support the use of evidence‐based therapies for diabetic foot infections while promoting antimicrobial stewardship, helping to prevent unnecessary broad‐spectrum or prolonged antibiotic treatments [42]. However, these guidelines, often developed by infectious diseases specialists based on clinical expertise and theoretical insights, may have limitations in practical application. They focus more on sharing knowledge rather than offering actionable strategies for implementing proven practices. As a result, many healthcare providers remain uncertain about managing complex infections, meeting patient expectations, balancing time constraints, and minimizing resource waste. Effectively addressing DFO requires a multidisciplinary approach, involving various specialists and nonphysician experts. Establishing a well‐resourced diabetic foot care team with dedicated time for its members is likely the most effective way to support both patients and healthcare providers.

Surgery plays a pivotal role in DFU management, ranging from simple ulcer debridement to revascularization procedures and amputations, either minor or major. In a cohort study by Aris et al [24], 72% of patients underwent minor (60%) and major (12%) lower limb amputations, exceeding the cumulative 54% observed by Richard et al [30]. They also demonstrated that only major amputations significantly decreased the time‐to‐heal of the wound, whereas minor amputations did not impact this timeframe [24]. This observation may be attributed to the persistence of infected bone or architectural reorganization of the foot, altering biomechanics and perpetuating a cycle of high‐pressure sites leading to skin breakdown and re‐ulceration [36]. A systematic review by Albright et al also demonstrated that a reduction of 39–56% for amputation rate can be expected when applying a multidisciplinary care team amputation prevention program [43]. Collaborative care involving endocrinologists, surgeons, infectious disease specialists, and podiatrists ensures comprehensive treatment, reduces complications, and enhances patient satisfaction. These findings suggest that conservative management of DFOM might, in some instances, only defer the inevitable, which is a major amputation.

The rising infection rates in individuals with diabetes, particularly in young adults, align with a recent upswing in diabetes‐related complications in the U.S. From 2010 to 2015, national data showed increases in lower‐extremity amputations and hyperglycemic crises, with stagnant progress in end‐stage renal disease, acute myocardial infarction, and stroke [44]. This surge in complication rates affects young (18–44 years) and middle‐aged (45–64 years) adults, with risks for hyperglycemic crisis, acute myocardial infarction, stroke, and lower extremity amputation each rising by over 25% in just 5 years. Gregg et al. [44] suggest several potential reasons for these trends. Firstly, the demographic profile of newly identified diabetes cases may be changing, with higher levels of obesity, smoking, and poor blood pressure and lipid management in younger adults with diabetes. Secondly, decreasing mortality and incidence of diabetes are extending the average duration of diabetes, potentially impacting complication risks [45]. Thirdly, a potential stagnation in preventive care, as evidenced by a decline in young adults meeting HbA1c targets, may contribute [45, 46]. Lastly, the introduction of high‐deductible health care plans and increasing costs of diabetes medications could be reducing early preventive care and potentially increasing risks of complications, including infections [46].

Our study has some limitations that warrant addressing. Firstly, it was conducted at a single referral high‐volume center within a limited time period. However, it is noteworthy that the number of cases in our study is comparable to findings in other reports. Additionally, our study lacks specific details on the causative agents, microorganisms involved in osteomyelitis, and their corresponding antibiotic treatments. We also did not collect the patient patients hematological and inflammatory. Also, while bone biopsy a more precise tool for the diagnosis of DFOM, MRI was used as the diagnostic tool due to practical limitations. While a history of prior wound infections was recorded as a variable, none of the patients included in the study had documented prior infections at the same site, other than the current diabetic foot ulcer episode under investigation. Despite these limitations, the identified risk factors in our study could offer valuable insights for health policymakers and aid in developing targeted group treatments and screening strategies to mitigate the risks associated with this hazardous complication.

## 5 Conclusion

As a conclusion, 23.8% of patients with a diabetic foot wound infection have osteomyelitis. In these patients, amputation can occur in 25%, wound dehiscence in 33.3%, recurrence of the wound in 30%, and death due to disease progression in 13.3%. In general, factors predicting the occurrence of osteomyelitis in the context of DFOM include lower age, lower BMI, higher income level, higher HbA1c level, and the location of the wound.

Overall, our findings highlight the significant burden of osteomyelitis in patients with DFUs, with significant implications for morbidity, mortality, and healthcare costs. Our study emphasizes the importance of early detection and effective management of risk factors to prevent the occurrence of this complication in DFUs.

## Author Contributions

**Soheil Bolandi:** methodology, data curation, writing – review and editing. **Mohammad Hadi Niyakan:** investigation, writing – review and editing. **Kazem Jamali:** conceptualization, writing – review and editing. **Yasaman Ghodsi Boushehri:** data curation, supervision, writing – review and editing. **Ali Tajaddini:** validation, writing – review and editing. **Reza Shahriarirad:** project administration, data curation, writing – original draft.

## Ethics Statement

The study was approved by the Research Ethics Committee of the School of Medicine‐Shiraz University of Medical Sciences (Ethical Code: IR. SUMS. MED. REC.1401.361). Permission to carry out the study and access patient records was sought from the respective university administrators, and the study was conducted in compliance in accordance with the relevant guidelines and regulations and the Declaration of Helsinki and was also approved by the ethics committee of the university.

## Consent

Written informed consent for participation was obtained from the patients.

## Conflicts of Interest

The authors declare no conflicts of interest.

## 1 Transparency Statement

The manuscript guarantor, Reza Shahriarirad, affirms that this manuscript is an honest, accurate, and transparent account of the study being reported; that no important aspects of the study have been omitted; and that any discrepancies from the study as planned (and, if relevant, registered) have been explained.

## Supporting information


**Supplementary Figure 1:** The MRI of the right foot in a patient with a diabetic foot ulcer shows no evidence of osteomyelitis or formation of collections.

**Supplementary Figure 2:** A multiplanar, multisequential MRI of the left foot reveals erosion and irregularity with hyperintensity in STIR sequences at the distal phalanx of the first toe, suggestive of osteomyelitis.

**Supplementary Figure 3:**The MRI of the left foot with and without contrast reveals evidence of a deep ulcer in the medial aspect of the plantar part of the left foot adjacent to the calcaneus, as well as another ulcer in the lateral aspect of the left foot associated with increased signal intensity in adjacent soft tissue and muscles, indicative of an inflammatory process such as cellulitis and myositis.

**Supplementary Figure 4:** Left foot MRI with and without contrast reveals a skin ulcer on the medial aspect of the calcaneus, measuring 10mm in length and 6mm in depth, accompanied by mild adjacent soft tissue edema.

## Data Availability

The data that support the findings of this study are available on request from the corresponding author. The data are not publicly available due to privacy or ethical restrictions.
