# Supplementary material for: Osteomyelitis and Its Main Determinants in Patients With Diabetic Foot Ulcer: A Cross‐Sectional Study
Source: Health Sci Rep. 2025 Nov 9;8(11):e71463. doi: 10.1002/hsr2.71463 (PMC12598195; doi:10.1002/hsr2.71463)
Supplement: Supplementary file 1 — Supplementary Figure 1: The MRI of the right foot in a patient with a diabetic foot ulcer shows no evidence of osteomyelitis or formation of collections. [file HSR2-8-e71463-s001.docx]

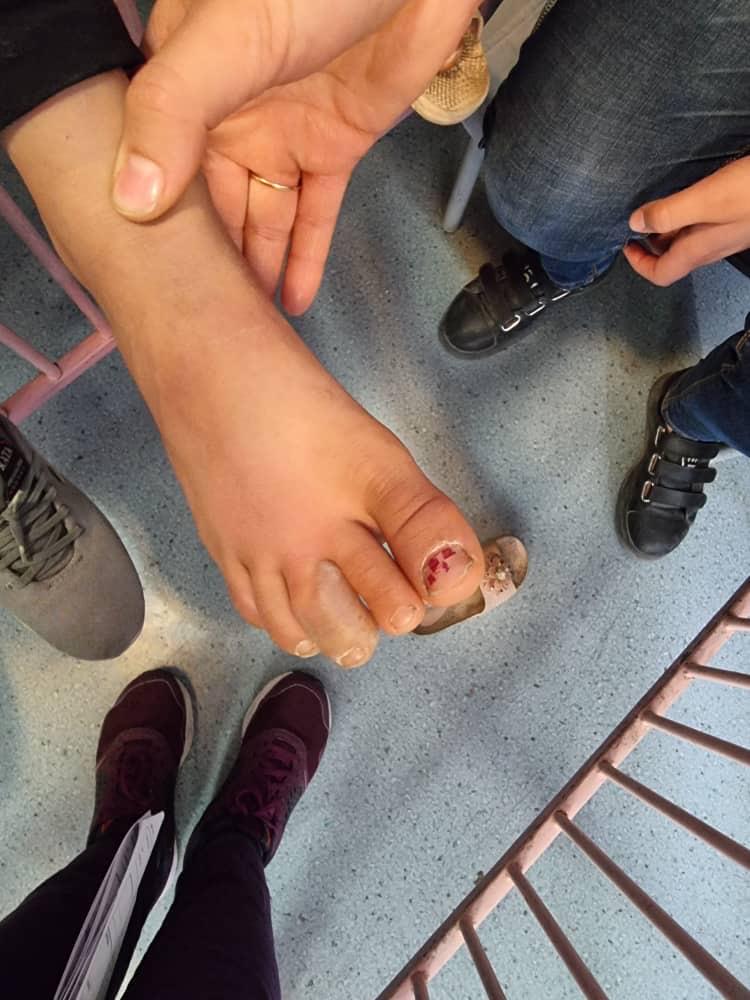

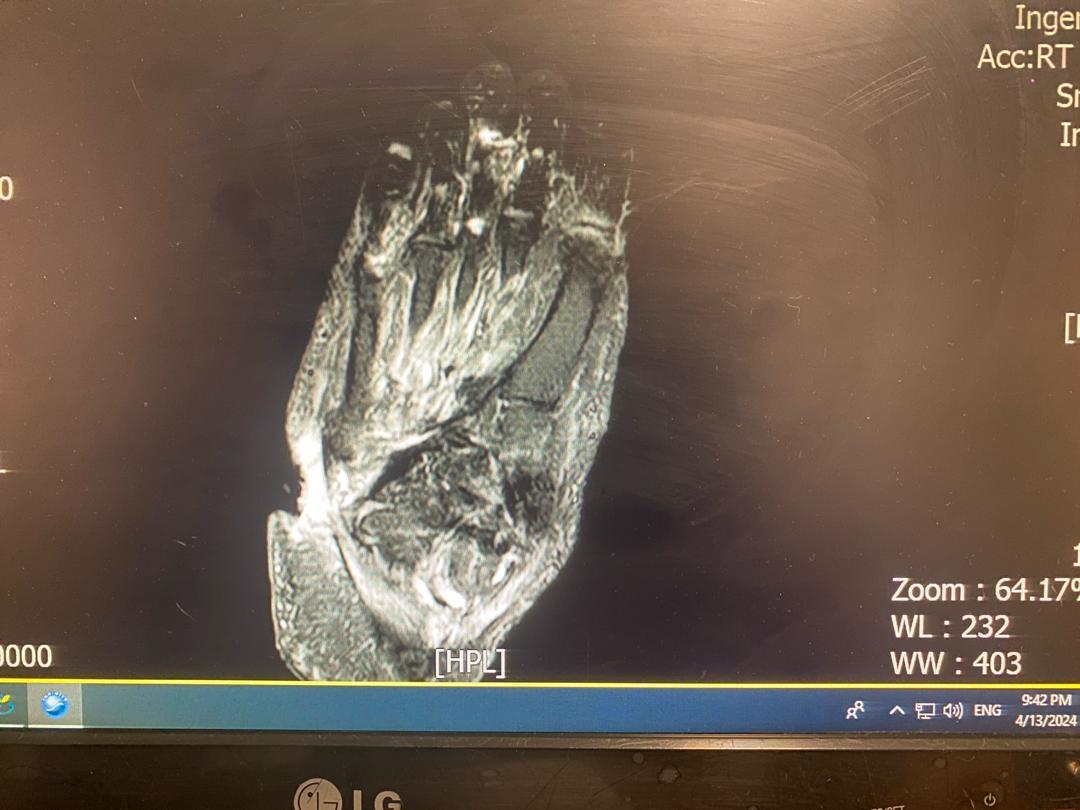

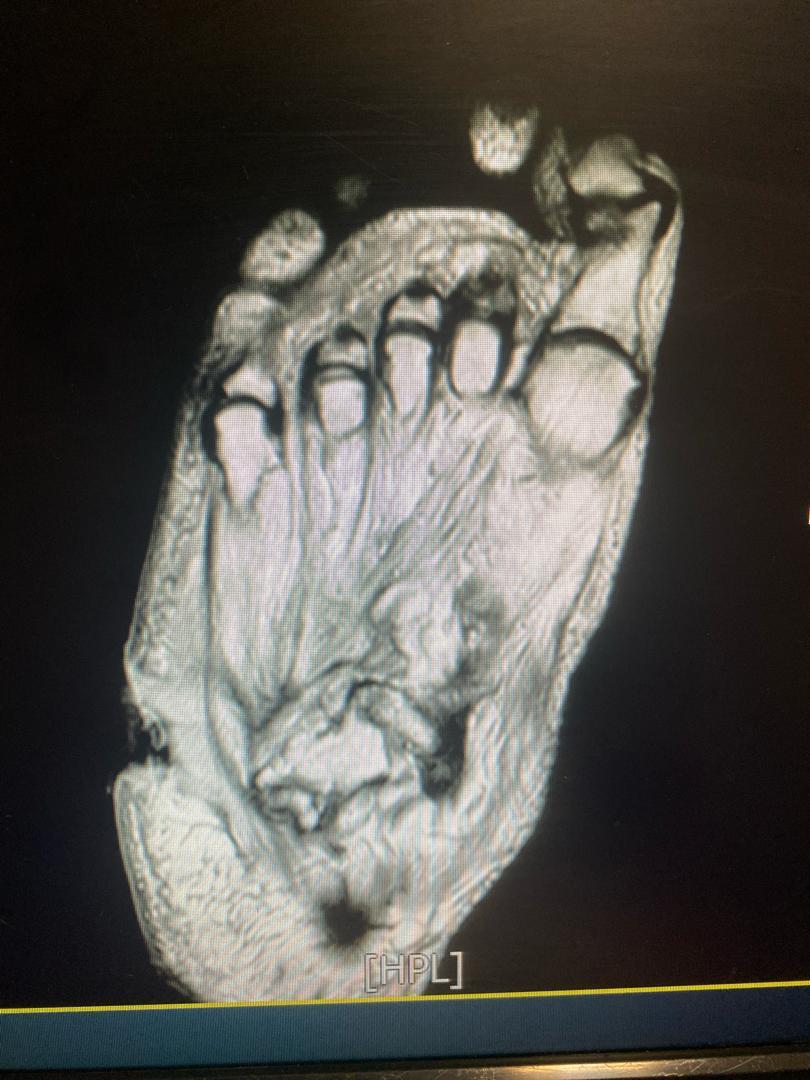


**Supplementary Figure 1.** The MRI of the right foot in a patient with a diabetic foot ulcer shows no evidence of osteomyelitis or formation of collections.
