# Supplementary material for: Osteomyelitis and Its Main Determinants in Patients With Diabetic Foot Ulcer: A Cross‐Sectional Study
Source: Health Sci Rep. 2025 Nov 9;8(11):e71463. doi: 10.1002/hsr2.71463 (PMC12598195; doi:10.1002/hsr2.71463)
Supplement: Supplementary file 2 — Supplementary Figure 2: A multiplanar, multisequential MRI of the left foot reveals erosion and irregularity with hyperintensity in STIR sequences at the distal phalanx of the first toe, suggestive of osteomyelitis. [file HSR2-8-e71463-s004.docx]

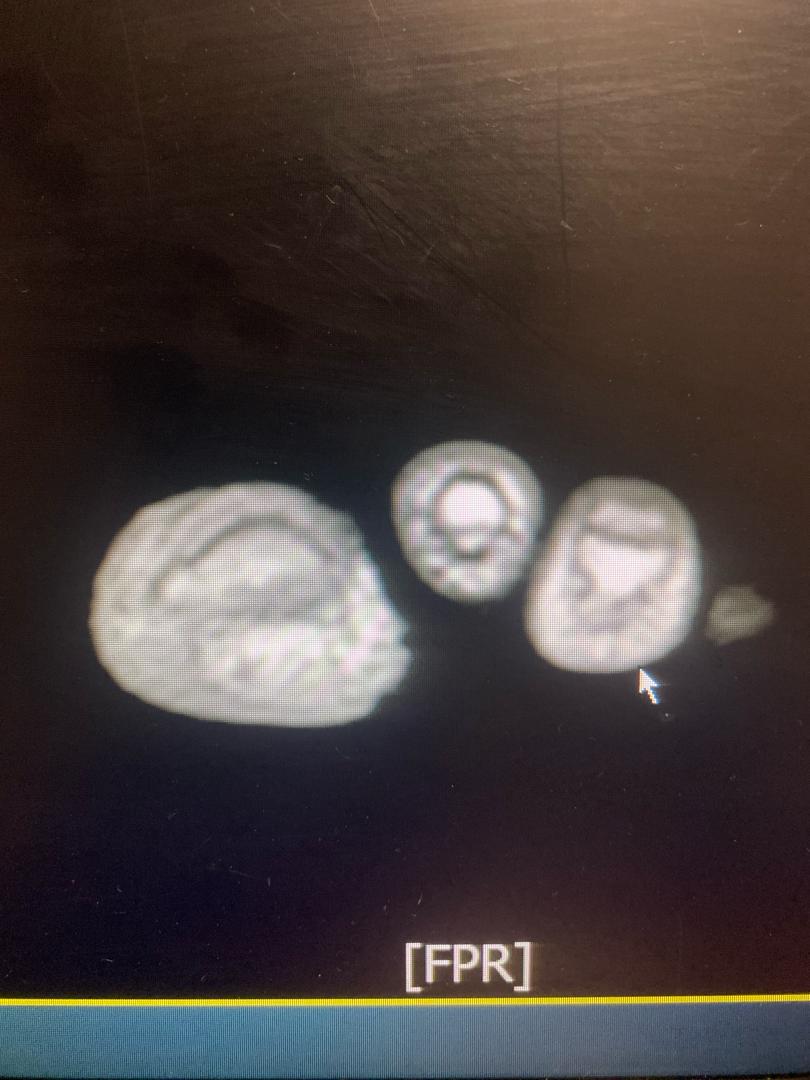

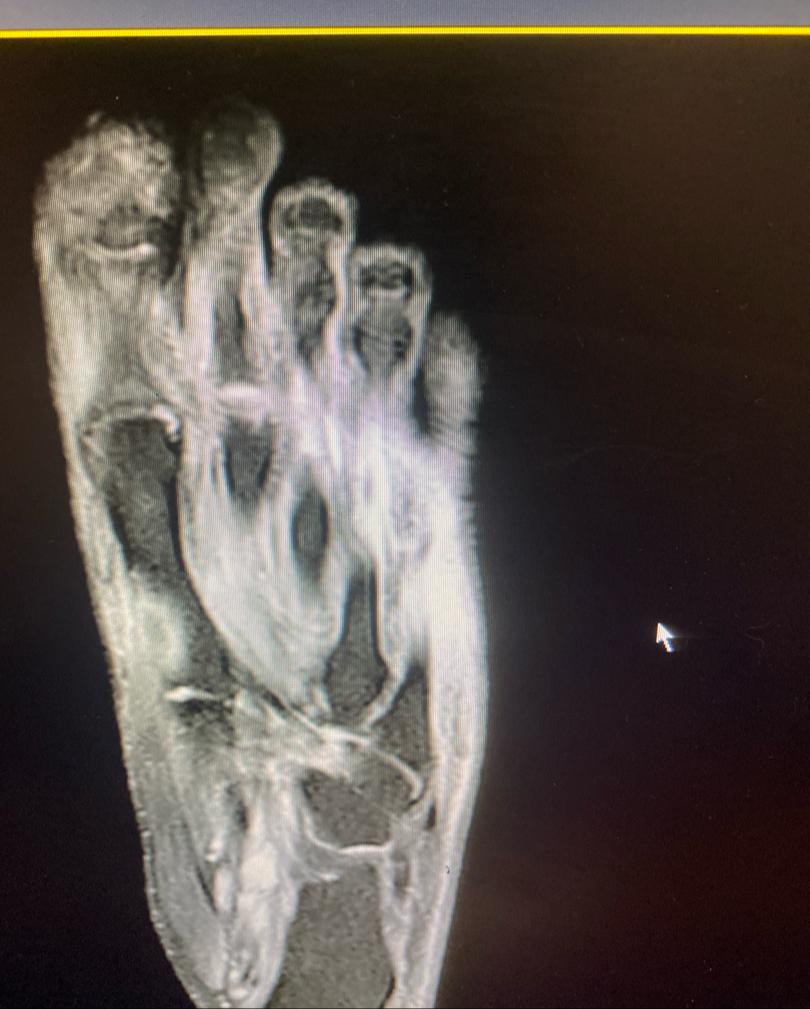

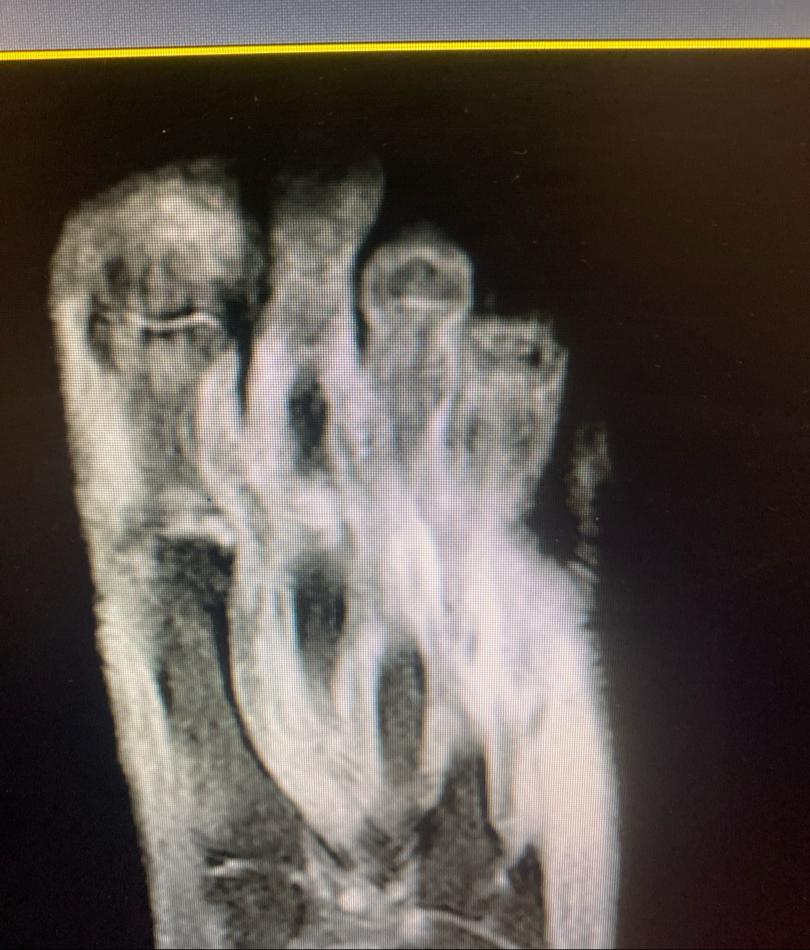

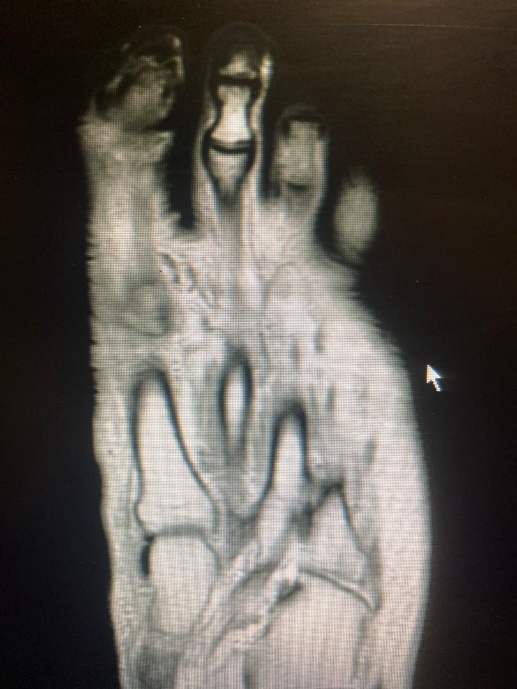


**Supplementary Figure 2.** A multiplanar, multisequential MRI of the left foot reveals erosion and irregularity with hyperintensity in STIR sequences at the distal phalanx of the first toe, suggestive of osteomyelitis. Significant soft tissue edema, particularly in the anterolateral aspect of the left foot, is observed. There is no evidence of collection formation or abscess. Other bony structures appear unremarkable.
