# Supplementary material for: Osteomyelitis and Its Main Determinants in Patients With Diabetic Foot Ulcer: A Cross‐Sectional Study
Source: Health Sci Rep. 2025 Nov 9;8(11):e71463. doi: 10.1002/hsr2.71463 (PMC12598195; doi:10.1002/hsr2.71463)
Supplement: Supplementary file 3 — Supplementary Figure 3:The MRI of the left foot with and without contrast reveals evidence of a deep ulcer in the medial aspect of the plantar part of the left foot adjacent to the calcaneus, as well as another ulcer in the lateral aspect of the left foot associated with increased signal intensity in adjacent soft tissue and muscles, indicative of an inflammatory process such as cellulitis and myositis. [file HSR2-8-e71463-s003.docx]

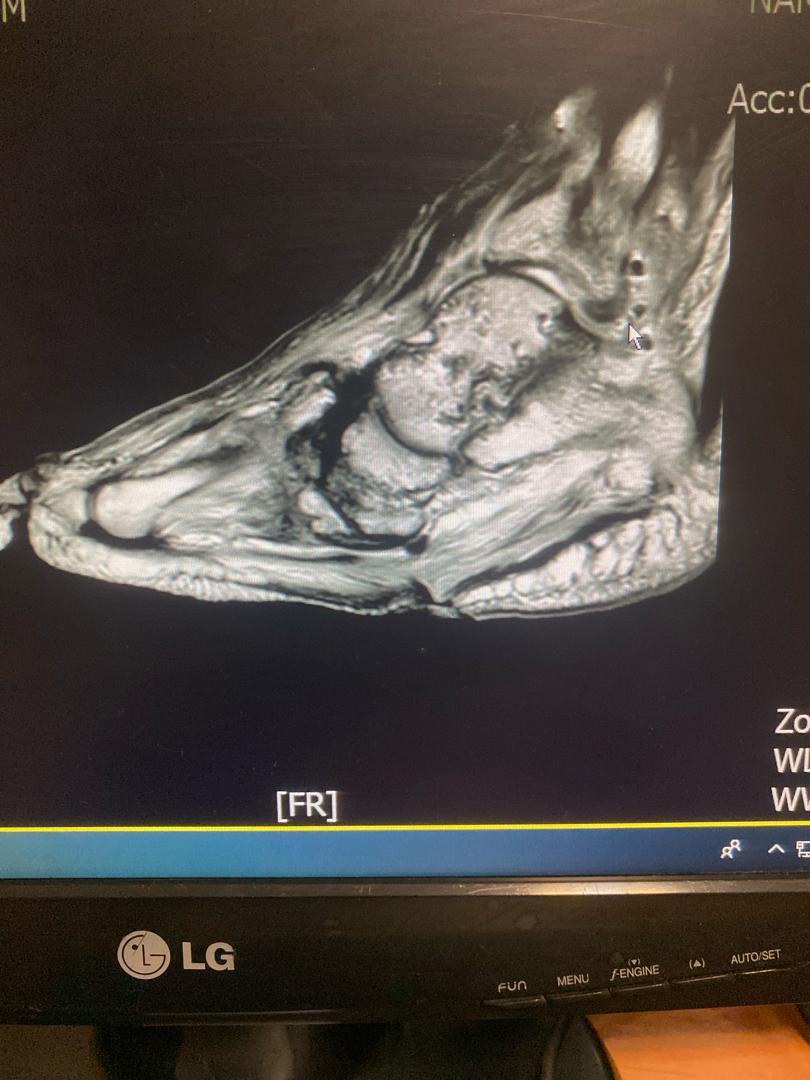

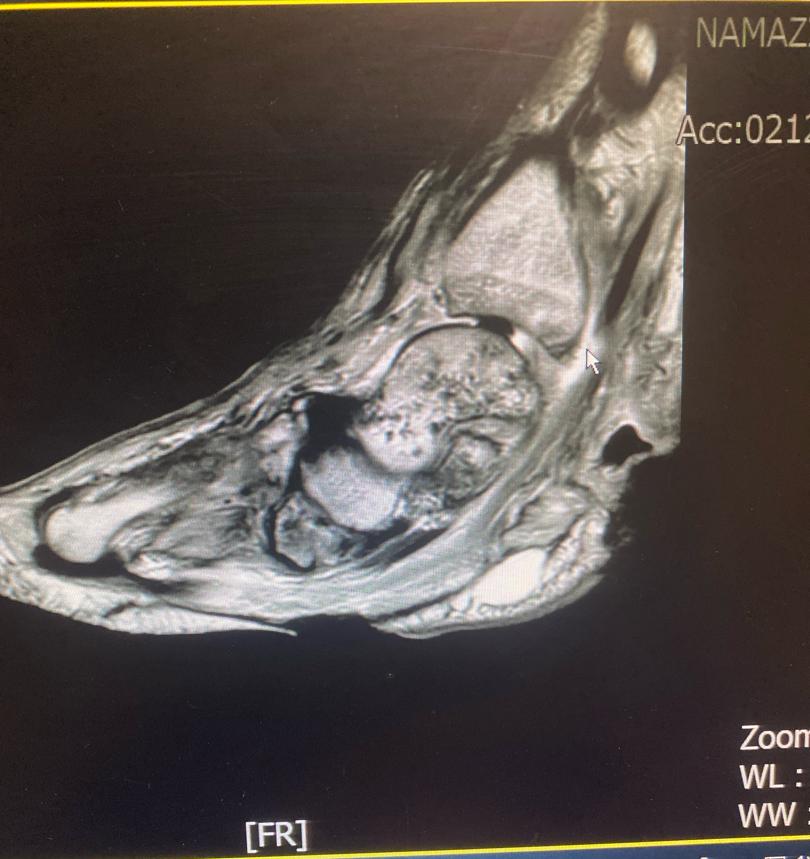

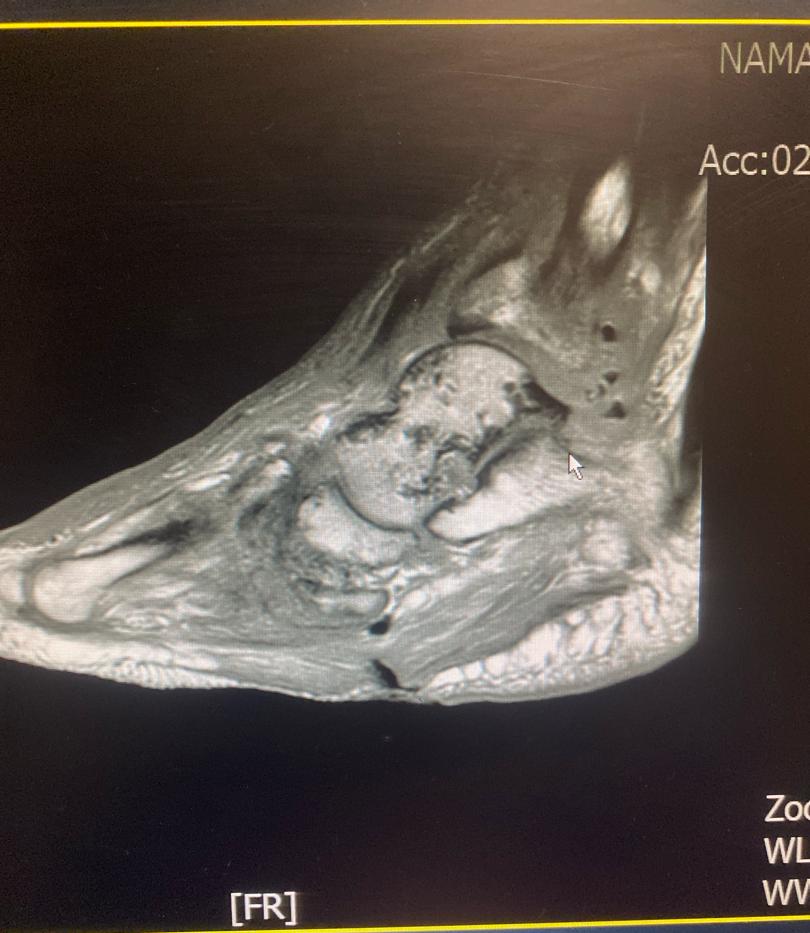

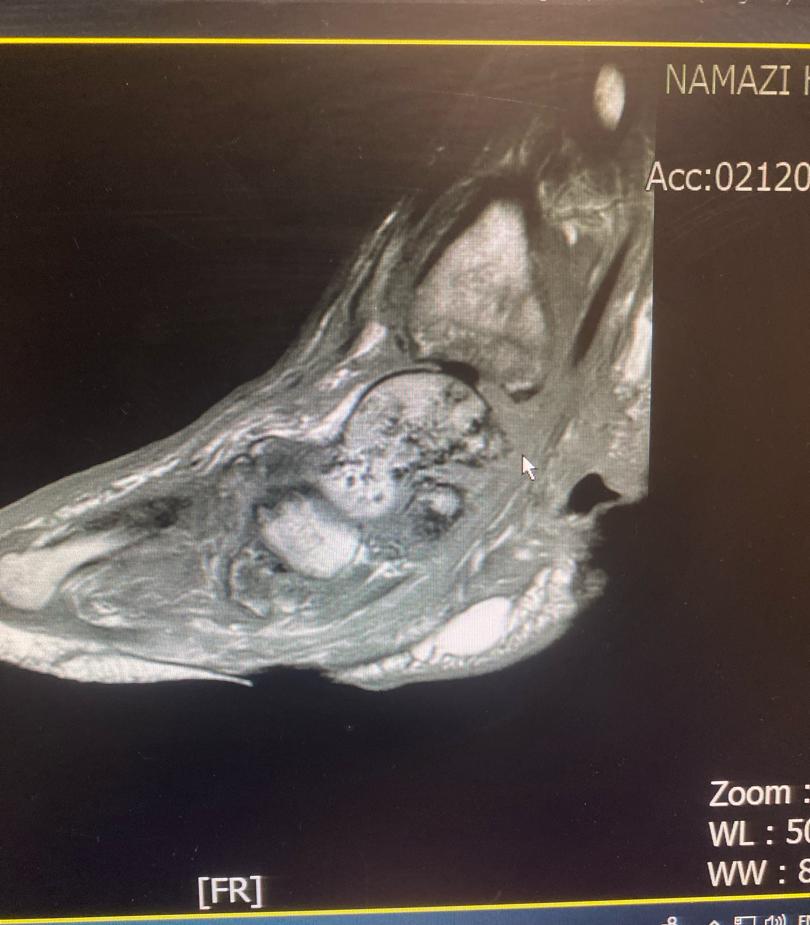


**Supplementary Figure 3.** The MRI of the left foot with and without contrast reveals evidence of a deep ulcer in the medial aspect of the plantar part of the left foot adjacent to the calcaneus, as well as another ulcer in the lateral aspect of the left foot associated with increased signal intensity in adjacent soft tissue and muscles, indicative of an inflammatory process such as cellulitis and myositis. Additionally, there is evidence of cortical bone irregularity, destruction, and decreased signal intensity in all tarsal and metatarsal bones (particularly in the tarsal bones and distal tibia), suggestive of Charcot joint and osteomyelitis. Synovial thickening and enhancement are observed in other metatarsal bones, along with bone marrow enhancement.
