# Supplementary material for: Osteomyelitis and Its Main Determinants in Patients With Diabetic Foot Ulcer: A Cross‐Sectional Study
Source: Health Sci Rep. 2025 Nov 9;8(11):e71463. doi: 10.1002/hsr2.71463 (PMC12598195; doi:10.1002/hsr2.71463)
Supplement: Supplementary file 4 — Supplementary Figure 4: Left foot MRI with and without contrast reveals a skin ulcer on the medial aspect of the calcaneus, measuring 10mm in length and 6mm in depth, accompanied by mild adjacent soft tissue edema. [file HSR2-8-e71463-s002.docx]

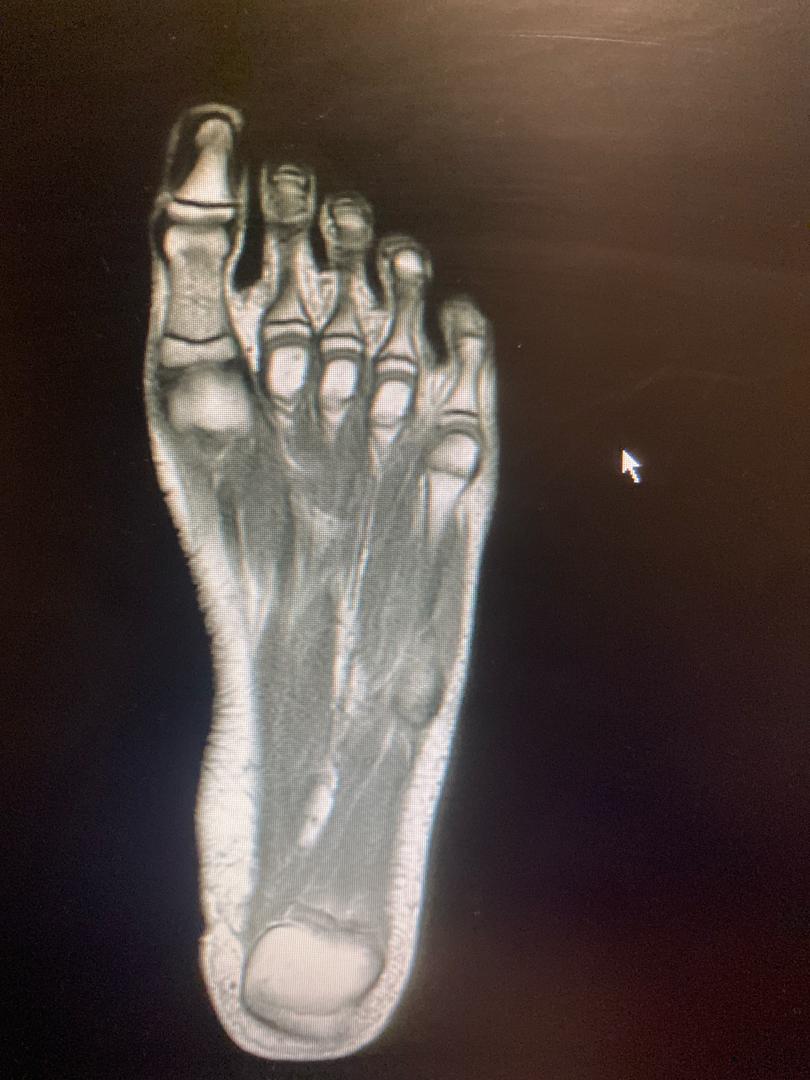

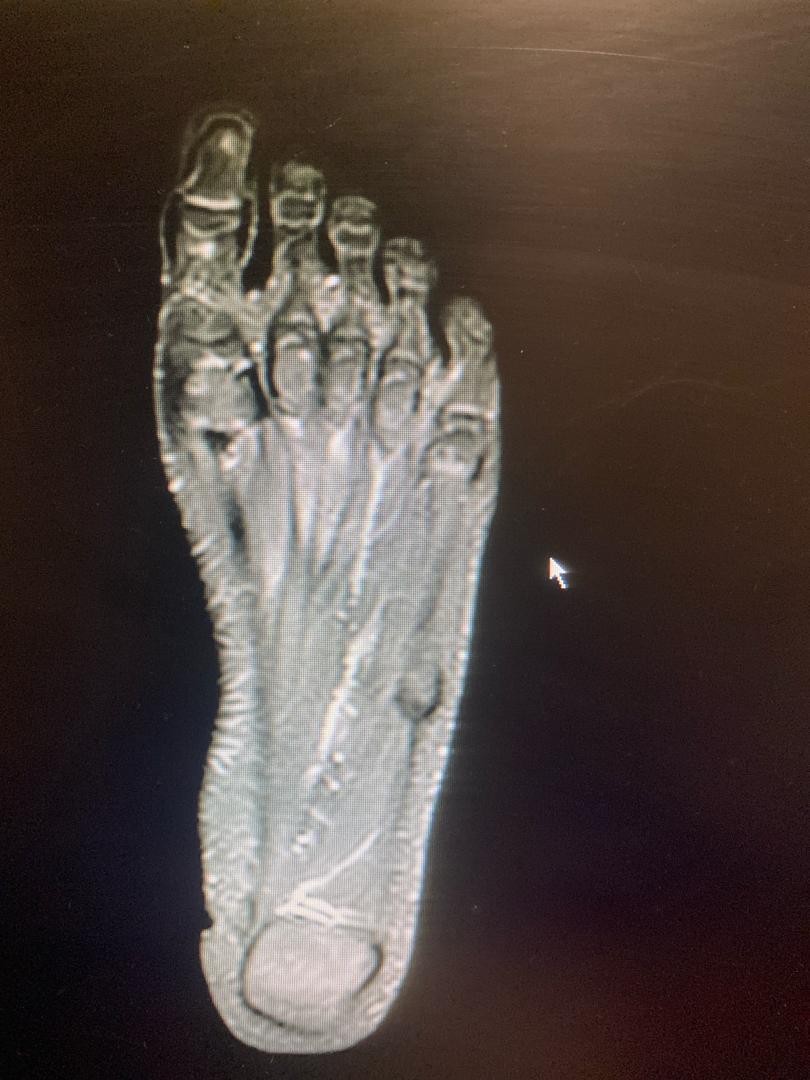


**Supplementary Figure 4.** Left foot MRI with and without contrast reveals a skin ulcer on the medial aspect of the calcaneus, measuring 10mm in length and 6mm in depth, accompanied by mild adjacent soft tissue edema. The scan shows significant synovial thickening and enhancement, as well as notable joint effusion. A small collection measuring approximately 12x5x-9mm is observed adjacent to the posterior distal end of the tibia. Multiple areas of patchy enhancement in all tarsal bones may correlate with systemic disease; further investigation correlating with past medical history is recommended. All visualized tendons and ligaments demonstrate normal signal intensity, with no signs of fracture or osteomyelitis detected.
